# Crossing the quality chasm in resource-limited settings

**DOI:** 10.1186/1744-8603-8-41

**Published:** 2012-11-30

**Authors:** Duncan Smith-Rohrberg Maru, Jason Andrews, Dan Schwarz, Ryan Schwarz, Bibhav Acharya, Astha Ramaiya, Gregory Karelas, Ruma Rajbhandari, Kedar Mate, Sona Shilpakar

**Affiliations:** 1 Nyaya Health, Bayalpata Hospital, Ridikot VDC, Achham, Nepal; 2 Brigham and Women’s Hospital, Department of Medicine, Boston, MA, USA; 3 Children’s Hospital of Boston, Department of Medicine, Boston, MA, USA; 4 Department of Psychiatry, University of California San Francisco, School of Medicine, San Francisco, CA, USA; 5 Institute for Healthcare Improvement, Cambridge, MA, USA; 6 Department of Medicine at Weill Cornell Medical College, New York, NY, USA; 7 Division of Infectious Diseases, Massachusetts General Hospital, Boston, MA, USA; 8 Global Health Equity Program Brigham and Women's Hospital and Children's Hospital of Boston, Boston, USA

**Keywords:** Resource-limited, Health system, Global health, Quality improvement

## Abstract

Over the last decade, extensive scientific and policy innovations have begun to reduce the “quality chasm” - the gulf between best practices and actual implementation that exists in resource-rich medical settings. While limited data exist, this chasm is likely to be equally acute and deadly in resource-limited areas. While health systems have begun to be scaled up in impoverished areas, scale-up is just the foundation necessary to deliver effective healthcare to the poor. This perspective piece describes a vision for a global quality improvement movement in resource-limited areas. The following action items are a first step toward achieving this vision: 1) revise global health investment mechanisms to value quality; 2) enhance human resources for improving health systems quality; 3) scale up data capacity; 4) deepen community accountability and engagement initiatives; 5) implement evidence-based quality improvement programs; 6) develop an implementation science research agenda.

## The quality chasm in resource-limited settings

A young school-age boy with severe respiratory distress presented to the remote hospital. Previously, the boy had been seen by untrained private clinicians in the community three times over four days. At presentation to the hospital, the child was evaluated by a mid-level practitioner who provided an initial course of antibiotics. Despite the child's ill appearance, supportive treatment including intravenous fluids and supplemental oxygen were not provided until discussion with the Medical Director three hours later. Later that evening, the electric nebulizer and oxygen concentrator became unusable after the hospital lost power owing to a blackout of the public electric grid and malfunctioning of the hospital’s backup generator. The regulator for the backup oxygen canister could not be found. At this juncture, without the ability to provide oxygen, the medical team recommended transfer. The family did not agree to transfer due to the high costs of other regional health facilities (our facility provides free services). That evening, after not being examined for over two hours by on-call staff, the child was found unresponsive with a thready pulse. Cardiopulmonary resuscitation was not initiated for over ten minutes as the midwife managing the ward did not know the procedure and the bag valve mask was not at the bedside. Following fifteen minutes of unsuccessful resuscitation, the child was declared dead.

Health care providers in resource-limited settings must do better [1]. Globally, resource-limited settings have received increasing funds over the last decade for the scaling up of health programs. These resources have generally been focused on the quantity of services provided. The quality of many of the resultant services, however, has often times been low or poorly understood. Since the 1980s, quality improvement (QI) research and implementation have taken steps towards reducing the “quality chasm” [2,3] that exists in developed-country settings [4-7]. If increased funding mechanisms for disease-focused global health initiatives was the major theme of the last decade in global health policy [8], then the next decade must emphasize the quality of health systems, and indeed several global initiatives are now underway [9,10]. Across all six domains of healthcare quality [2] - safety, effectiveness, efficiency, patient-centeredness, timeliness, and equity-bridging the global quality chasm requires reconceptualizing the delivery of health services to the global poor. The experiences of clinicians delivering health care in rural regions have made clear the urgent need for new strategies. These strategies must address logistical, supply chain, engineering, human resources and traditional clinical challenges, while remaining cognizant of and adaptable to the unique medical and socio-cultural needs of each setting. While there are many local and global initiatives currently ongoing in this area, there is a need for a concise vision from the view of grassroots providers. Towards this end, this perspective paper describes one vision for a global quality improvement movement (Additional file 1: Figure S1.

If such a movement were to take hold, how might the young school-age boy have fared differently? Let us re-envision the case with a different outcome, one that we propose a global QI movement could achieve. One day after his parents reported a cough and fever, the boy was visited by a community health worker and referred to a health post where he received an initial course of antibiotics. On the second day, when follow-up revealed a higher fever and respiratory distress, the child was referred to the hospital. After initial consultation, a mid-level provider admitted the patient and spoke directly with the Medical Director to ensure proper management. Oxygen was administered via a generator-powered nebulizer, and on the third hospital day, after significant decline, the child was transferred via an oxygen-equipped ambulance to a regional hospital, where intensive care unit staff were expecting the referral. After eight days on the inpatient unit at the regional hospital, the child followed up as an outpatient and was visited at home regularly by a community health worker. The following week, the family attended a community health worker class on respiratory illness and prevention in the community.

### A global quality improvement movement

The following broad categories will be the pillars of an effective global QI movement: 1) revise global health investment mechanisms to value quality; 2) enhance human resources for improving health systems quality; 3) scale up data capacity; 4) deepen community accountability and engagement initiatives; 5) implement evidence-based QI programs; 6) develop an implementation science research agenda.

### Revise global health investment mechanism to value quality

The dominant approach to global health financing remains tied to volume-based targets, such as the number of patients provided family planning services or patients initiated on antiretroviral treatment [11]. Emphasizing quality metrics in the planning and financing of health services could alter the incentives of health systems actors to provide better quality, not just quantity, healthcare. Several individual projects and financing schemes have developed quality metrics, but a more concerted, cultural shift is required [11,12]. Investments in quality health systems will take longer to bear fruit, so donors will have to adapt their mindsets and establish interim benchmarks for longer-term objectives. In the case of this hospital, we would have access to additional training programs, and would be monitored by district authorities and local community development committees to improve quality, if central funding mechanisms were revised with an eye towards quality.

### Enhance human resources for improving health systems quality

Global healthcare faces crisis-level personnel shortages, which make developing and sustaining QI initiatives extremely difficult [13]. In addition to recruitment and retention initiatives, the development of a global QI movement will require further focus on QI-related medical education. Since many developing nation medical education centers are currently unequipped to effectively teach QI [14,15], developing QI-related curricula, continuing medical education programs and regular competence evaluations and self-assessments for all levels of healthcare providers [16-18] will be critical. Moreover, a particular focus on rural facilities where personnel have little, if any, interaction with academia or continuing educational efforts would be important. QI initiatives also lead to improved system performance and efficiency improving health workforce productivity and boosting morale.

In the case of the young school-age boy, more robust QI-related education during staff training, and regular staff competence review in our hospital could have identified and addressed many of the issues that resulted in this boy’s death. For example, performance evaluations of our nurse midwives would have documented insufficient knowledge of resuscitation protocols, enabling targeted education to improve resuscitation quality.

### Scale up data capacity

Health systems learn much like people do by receiving constructive and context-specific feedback on their performance. As such, a culture of real-time data collection and evaluation is crucial to the QI endeavor [19]. Data systems are difficult to establish in resource-limited settings, due to low computer literacy, unreliable electricity, and competing financial needs that supercede information technology investments. Basic reporting schemes are often incomplete or inaccurate, and are not analyzed in a provider-friendly format. Moreover, most reporting systems rely on inefficient paper systems and lack the infrastructure to upgrade to electronic versions [20]. Importantly, most clinics and health care providers who generate data for public health reporting rarely receive feedback on their performance. For information to drive clinical practice, data systems need to be timely, accurate, accessible, and relevant. They need not and should not be particularly complex. To avoid duplication with existing national data systems, the collection and uses of data should be reviewed and QI metrics should be incorporated within national data systems. These data systems should be developed to feedback to providers with information on their performance, comparison to other facilities, and areas for improvement.

In the case of the young school-age boy, data on pneumonia treatment in our facility will help inform the clinical practice and process improvements we make. To evaluate our pneumonia management we will need to know, for example, rates of febrile children for whom a respiratory rate was documented, action taken based upon established protocols (e.g., Integrated Management of Childhood Illness [21]), and rates of appropriate antibiotic prescription.

### Deepen community accountability and engagement initiatives

Accountability practices have been shown to enhance QI in numerous settings, yet are significantly lacking globally. In many resource-limited settings, low education rates paralleled by unequal power dynamics between health providers and patients make accountability challenging. Strategies to address these issues include holding public meetings about available services [22], making performance data publicly available [23], community-accountability checklists, and publishing health institution report cards [24].

All of these serve to engage patients and community members in taking ownership over quality of healthcare for their citizens. Community groups will need to be supported with both financial and technical assistance to ensure that they have the resources to provide excellent monitoring of the quality of the healthcare they receive. The overall goal is to inform the communities each health facility serves, augmenting efforts for constant service improvement and informed community decision making. In the case of the young school-age boy, knowing the performance metrics of untrained care providers might have influenced his parents to bring him directly to the hospital, before his condition had reached an emergency level.

### Implement evidenced-based quality improvement programs

There is an acute need to close the "implementation gap" in global healthcare delivery [25,26]—that disconnect between what is known and what is actually available in resource-limited settings. Some approaches unique to resource-limited environments may be needed, but while we await the research and development of such strategies, successful QI paradigms utilized in resource-rich contexts could be adapted and implemented. The following non-exhaustive list outlines a few simple ways to implement QI practices within diverse and resource-limited settings.

The Plan-Do-Study-Act (PDSA) model is a component of the well-tested Model for Improvement. The PDSA could help meet the lack of critical reflection and operations improvement within global health [27]. Within PDSA, straightforward cause-and effect process mapping through Ishikawa or other diagrams could engage providers at all educational levels, and enable critical staff buy-in within resource-limited healthcare institutions. This model can be applied in the clinical, outreach, managerial, logistical, supply chains, and engineering realms of healthcare management. Other QI tools like the Breakthrough Series College can assist program designers and health systems planners to more rapidly spread best practice throughout entire districts and even countries [28,29].

Checklists designed to ensure standardized, high quality care and team communication improve the effectiveness of clinical care in resource-limited settings [4,30]. Checklists are a low-tech solution that can bolster managerial oversight and better utilize available human resources, improving overall effectiveness. Checklists are an important tool for standardization but their effective implementation will have to undergo rigorous site-specific testing using QI principles.

Morbidity and mortality (M&M) case conferences can ensure both ongoing clinical education and regular, inclusive, team-based process evaluation [31]. This has been our experience in rural Nepal, where health personnel typically have few opportunities for continuing medical education or team-based reflection. M&Ms in other locations have also been transformed into QI meetings where district/subdistrict process and outcome data are shared with a community of clinics and hospitals to assist with data feedback.

Multidisciplinary care teams for patients with chronic medical conditions have also improved quality in diverse settings [32,33]. Community health workers should form the foundations of these teams [34]. Much of the primary care system infrastructure in developing countries was designed to manage acute ailments and functions as a network of urgent instead of primary care centers. Multidisciplinary teams can help change this dynamic.

If implemented broadly and adapted appropriately, such strategies would ultimately offer a rubric of standard high quality healthcare practices to diverse resource-limited environments. Many of the errors that occurred in our patient's care could have been avoided by applying basic, low-tech practices such as these.

### Develop an implementation science research agenda

An implementation science research agenda is crucial to advancing the field and practice of quality improvement. Research into quality in global health has historically focused on the efficacy of interventions demonstrated through large-scale randomized controlled trials and subsequent guideline development. The new implementation science research agenda must focus on understanding implementation in context which requires new context-specific evaluation tools like time-series analysis, statistical process control or adaptive clinical trials. Also, the areas of investigation should not center on new therapeutic strategies tested in controlled environments but the application of known strategies in diverse, uncontrolled environments.

Furthermore, research is needed not just on clinically-focused problems but also on logistical and managerial ones [35,36]. Waste, water quality, energy, infectious disease control, supply chains, human resource management systems all require study, reflection, and scrutiny. This research should be coordinated, tied to real practice, and focused on both outcomes and processes. A central research methodology will include in-depth qualitative and descriptive quantitative analyses of the implementation of and longitudinal follow-up of individual QI projects. This should be supplemented by more costly randomized controlled trials comparing QI interventions and non-randomized multi-site outcomes-oriented longitudinal studies. Through such research, we can help rectify the logistical, communication, and managerial failures that led to the death of the young school-age boy.

### A global quality improvement movement

With the aim of achieving improved outcomes for patients globally, we have underscored the importance of a global healthcare QI movement and outlined initial steps toward it. The far-reaching goals of global health do not stop with health systems scale-up. Rather, scale-up is the foundation of a larger movement aimed at delivering quality healthcare services for all. To achieve this vision, we will need new resources and a concerted, quality-oriented agenda. Many of these interventions will be relatively inexpensive, though data on the cost and effectiveness of these interventions is currently unknown; as the movement develops, efforts will be needed to centralize these data to ensure cost-effective use of resources.

We can save lives and prevent disability by improving the quality of healthcare services in the resource-limited settings. The immediate actions described in this article towards achieving healthcare quality improvement are important. It may be overwhelming and impractical to achieve all quality improvement measures in a short time. However, if actions are taken, quality improvement can be achieved in the long term. Collective efforts from funding sources as well as healthcare consumers would help to achieve this common goal of healthcare quality improvement in resource-limited settings.

## Abbreviations

QI: Quality improvement; PDSA: Plan-Do-Study-Act; M&M: Morbidity and mortality.

## Competing interests

The authors declare that there is no financial or non-financial competing interest.

## Authors’ contributions

The authors are professionals who work in varying capacities with Nyaya Health, a non-profit group delivering healthcare services in rural Nepal that is innovating methods of open-access and transparency in the field of global health. Together, they have conceptualized this piece as a reflection upon their collective experiences within Nepal and individual ones in other resource-limited areas. All authors reviewed and provided inputs and edits to the final piece. DM conceived the piece, wrote the initial draft, and serves as the guarantor of the article. All authors read and approved the final manuscript.

## Authors’ information

Authors are affiliated with the Bayalpata Hospital which is located in far west of Nepal. The case presented in this article was the case encountered at the Bayalpata Hospital. Based upon author`s experiences delivering health care in resource limited-setting, authors propose the actions that needs to be taken to achieve healthcare quality improvement in resource-limiting settings.

## Supplementary Material

Additional file 1 **Figure S1.** Strategies for a Global Quality Improvement Movement. Click here for file
