# Supplementary material for: Crossing the quality chasm in resource-limited settings
Source: Global Health. 2012 Nov 30;8:41. doi: 10.1186/1744-8603-8-41 (PMC3526495; doi:10.1186/1744-8603-8-41)
Supplement: Additional file 1 — Figure S1. Strategies for a Global Quality Improvement Movement. [file 1744-8603-8-41-S1.doc]

**Additional file 1: Figure S1 Strategies for a Global Quality Improvement Movement**

| Revise global health investment mechanism to value quality |
| --- |
| Enhance human resources for improving health systems quality |
| Scale up data capacity |
| Expand accountability practices |
| Implement evidenced-based quality improvement programs |
| Develop an implementation science research agenda |
